# Neonatal Kawasaki disease with multiple arterial aneurysms: a case report

**DOI:** 10.1186/s12969-020-00440-x

**Published:** 2020-06-15

**Authors:** Qu-ming Zhao, Xue-cun Liang, Lin Wu, Fang Liu

**Affiliations:** Pediatric Heart Center, Children’s Hospital of Fudan University, 399 Wan Yuan Road, Shanghai, 201102 China

**Keywords:** Neonate, Kawasaki disease, Coronary artery aneurysms, Systemic artery aneurysms, Fever

## Abstract

**Background:**

Kawasaki disease (KD) is a medium vessel vasculitis that typically occurs in children aged between 6 months and 5 years. It is extraordinarily rare in the neonatal period. KD-related systemic artery aneurysms (SAAs) have never been reported in neonates.

**Case presentation:**

A male infant was transferred to our institution for persistent high-grade fever lasting 16 days. Symptoms started at day 14 of life, and he was admitted to a children’s hospital on the second day of fever. Physical examination at the time found no signs suggestive of KD. The only laboratory parameters which were of significance were values suggestive of systemic inflammation. However, his fever persisted and inflammatory markers continued to rise despite 2 weeks of antibiotic therapy. KD as a noninfectious cause of fever was considered when he came to our institution, and echocardiographic findings of left and right medium coronary artery aneurysms (CAAs) confirmed our suspicions. Full-body magnetic resonance angiography also revealed bilateral axillary artery aneurysms. Administration of intravenous gamma globulin resulted in rapid improvement. His fever resolved on the next day and CAAs and SAAs regressed to normal at 6 months and 3 months after diagnosis, respectively.

**Conclusion:**

This unique case of incomplete KD highlights the importance of considering KD in neonates with unexplained prolonged fever and reinforces the need to remain vigilant for SAAs in KD.

## Background

Kawasaki disease (KD) is a self-limiting systemic vasculitis of unknown etiology that typically occurs in children aged between 6 months and 5 years [1]. It is much less common under 3 months of age and extraordinarily rare in the neonatal period [2–8]. A 12-year Japanese nationwide survey reported only 23 cases of neonatal KD [2], while only about 10 neonatal cases have been reported in other countries in the English-language literature [4]. Neonatal KD is uncommon, and as such when cases do arise, it is important that they are shared so that general pediatricians and neonatologists are able to recognize this presentation, especially in very young infants [9]. When misdiagnosed as other infectious diseases, affected children are at risk for delayed diagnosis and coronary artery aneurysms (CAAs) [10]. KD-related systemic artery aneurysms (SAAs) are currently thought to be not uncommon [11] but have never been reported in neonates. Here we report a case of delayed diagnosis of neonatal KD with both coronary artery and axillary artery aneurysms.

## Case presentation

A 30-day-old male infant was transferred to our institution for persistent high-grade fever lasting 16 days. Symptoms started on day 14 of life, and he was admitted to a tertiary-level children’s hospital on the second day of illness, at which time he had no skin, respiratory, gastrointestinal, or nervous system symptoms. Admission laboratory tests revealed a normal complete blood count, serum transaminase levels, albumin, antinuclear antibodies, immunoglobulin levels, and CD markers, but elevated C-reactive protein (CRP) (50 mg/L), erythrocyte sedimentation rate (ESR) (55 mm/h), ferritin (348 ng/ml) and procalcitonin (0.96 ng/ml). His chest X-ray and abdominal ultrasound were unremarkable. Empirical antibiotic therapy comprising of ampicillin and cefotaxime was started for presumed neonatal sepsis. Physical examination was within normal limits except for a transient day-long generalized reddish rash and mild conjunctival congestion on day 6 of fever, which was considered by the neonatologist to be a manifestation of infection. However, bacterial cultures of blood, urine, stool, and cerebrospinal fluid, as well as viral screens for toxoplasmosis, rubella, cytomegalovirus, herpes simplex, adenovirus, respiratory syncytial virus, Influenza A and B, Epstein Barr virus, and rotavirus were all negative. Unfortunately, his fever persisted even after antibiotics were upgraded to vancomycin and meropenem.

**Fig. 1:**
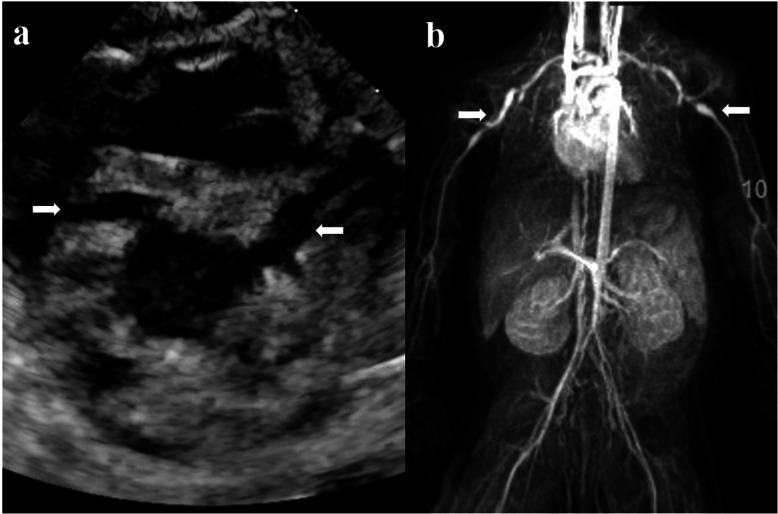
**a** Echocardiography at 2 weeks after the onset of KD revealed medium coronary artery aneurysms (arrow) in both the left and right coronary arteries. **b** Magnetic resonance angiography at 2 weeks after the onset of KD revealed bilateral axillary artery aneurysms (arrow)

By the time he was admitted to our hospital, his white blood cells, platelets, CRP and ferritin had risen to 26.8 × 10^9^/L, 470 × 10^9^/L, 160 mg/L and 595 ng/ml, respectively. In contrast, his procalcitonin had decreased to 0.50 ng/ml, while at the same time having hypoalbuminemia (25 g/L) and anemia (95 g/L). At this point, as no clear etiological evidence was found, KD as a noninfectious cause of fever was the first to be considered according to the 2017 American Heart Association (AHA) guidelines [12]. On day 2 of admission, echocardiographic findings of the left anterior descending artery (LAD) and right coronary artery (RCA) revealed medium CAAs, confirming our suspicions (Fig. 1). The internal diameter of the LAD and RCA were 3.5 mm (z score = 6.7) and 2.9 mm (z score = 5.8), respectively. Full-body magnetic resonance angiography (MRA), performed routinely in patients with medium to giant CAAs in our institution [11], also revealed bilateral axillary artery aneurysms that could not be palpated on physical examination (Fig. 1). Syphilis, which can also cause multiple aneurysms, was unlikely given a negative rapid plasma regain test and Treponema pallidum particle agglutination test. Considering the early age of onset, we also did whole-exome sequencing to identify mutations in known candidate genes (such as Adenosine deaminase 2 gene) or other unknown genes which may have a potential role in the development of this presentation.

Intravenous gamma globulin (IVIG) (2 g/kg), methylprednisolone (2 mg/kg.d), aspirin and low molecular weight heparin (LMWH) (75u/kg.dose q12h) were administered immediately. The next day, his fever resolved and his CRP level began to decrease, and 1 week later, slight periungual desquamation of the fingers and toes was noted. Subsequent echocardiographic follow-up revealed no worsening of the coronary lesions. On day 30 of admission, glucocorticoids were stopped, and he was discharged home on warfarin and aspirin. Three months after diagnosis, echocardiography showed that the diameter of the LAD and RCA had been reduced to 2.3 mm (z score = 2.3) and 2 mm (z score = 2.1), respectively, and MRA showed complete resolution of the axillary artery aneurysms. Warfarin was thus discontinued. Gene sequencing revealed no gene mutations associated with his symptoms. Aspirin was stopped 6 months after diagnosis, by which time the diameter of the coronary arteries had returned to normal.

## Discussion

Both neonatal KD and KD-related SAAs are not well recognized due to their rarity, and thus there are only sporadic reports of a few cases in the English language literature concerning either of these issues [13–16]. To the best of our knowledge, this is the first report of KD in a newborn with both CAAs and SAAs.

Extremes of the pediatric age range represent a significant risk factor for the development of CAAs and incomplete presentation [9]. A review of the literature revealed that 56.3% (9/16) of reported cases of neonatal KD have been associated with CAAs, and 75% (12/16) have an incomplete presentation [4]. Current clinical criteria may not always identify KD in young infants in a timely fashion [10]. The clinical symptoms in young infants with KD, as in our case, can be short-lived and thus easily misdiagnosed as a transient manifestation of an infectious disease. It should be emphasized that echocardiography needs to be performed in febrile young infants who do not respond to antibiotic therapy despite incomplete manifestations, as timely diagnosis and treatment are essential for patients with KD to reduce the risk of cardiac complications. Although the uncommon presentation of KD in this newborn may have prompted clinicians to consider other medium vessel vasculitides such as polyarteritis nodosa, the efficacy of IVIG and favorable prognosis led us to believe that KD was the most appropriate diagnosis for this patient. In terms of initial therapy, corticosteroids are often used in addition to IVIG in high-risk patients or those with CAAs at diagnosis, and they have been proven to have a favorable long-term effect on CAAs [17]. Considering the early age of onset and multiple aneurysms in this patient, anticoagulation with LMWH or warfarin was added to aspirin for more aggressive prophylaxis of thrombosis, which is also a class IIb recommendation of the 2017 AHA guidelines for medium CAAs [12].

A previous study by our team demonstrated that the incidence of SAAs in KD is not as low as we thought. Longer duration of fever, larger CAAs, and younger age may be risk factors for SAAs, with the regression rate of SAAs was better than that of CAAs over time [11]. The clinical features and prognosis of this newly identified patient are consistent with our previous findings, and again suggest that clinicians should be alert to the possibility of SAAs in KD, as large SAAs may progress to obstructive lesions or cause ischemic symptoms. Based on our preliminary experience, we suggest that full-body MRA screening may be indicated in young infants or patients with medium to giant CAAs, but more data are needed to confirm this speculation.

## Conclusion

This rare case of incomplete KD highlights the importance of considering KD in neonates with unexplained prolonged fever, who are more likely to present with incomplete KD and coronary artery lesions. SAAs are another essential clinical problem that should not be overlooked in KD, and research is needs to explore this issue.

## Data Availability

Not applicable.

## Abbreviations

CAAs: Coronary artery aneurysms
CRP: C reactive protein
IVIG: Intravenous immunoglobulin
KD: Kawasaki disease
SAAs: Systemic artery aneurysms
